# The structural characterization of a polysaccharide exhibiting antitumor effect from *Pholiota adiposa* mycelia

**DOI:** 10.1038/s41598-018-38251-6

**Published:** 2019-02-11

**Authors:** Yajie Zou, Fang Du, Qingxiu Hu, Hexiang Wang

**Affiliations:** 1 Institute of Agricultural Resources and Regional Planning, Chinese Academy of Agricultural Sciences, 12 Zhongguancun South Street, Beijing, 100081 China; 2 State Key Laboratory for Agrobiotechnology and Department of Microbiology, China Agricultural University, 2 Yuanmingyuan West road, Beijing, 100193 China

## Abstract

PAP80-2a, purified from *Pholiota adiposa* mycelia, is a polysaccharide exhibiting prominent antitumor effects. However, the yield of PAP80-2a was low and its structure has not been characterized, impeding the exploration of its structure-function relationship, thus influencing the development of oral drugs for antitumor therapy and immunomodulation. In order to improve the yield of PAP80-2a, response surface methodology along with Box-Behnken design was applied to optimize the ultrasonic-assisted extraction conditions for polysaccharides. Then, the structure of PAP80-2a exhibiting antitumor activity was determined from different angles. The results showed that the extraction yield of *P. adiposa* polysaccharides increased by 11.5% under optimized ultrasonic extraction conditions. Structural analysis showed that PAP80-2a was mainly composed of glucose, rhamnose, xylose, and galactose in a ratio of 10.00: 2.09: 4.09: 1.13. The total amino acid content in the sugar chain was 69.92 μg/mL. The sugar chain structure was [α-Rha (1 → 3)-]n, and rhamnose was located at the non-reducing end of the sugar chain, while glucose was located at the non-reducing end or in the sugar chain in 1,2,6- and 1,3,6-linked forms. Our study clearly illuminates the primary structure of PAP80-2a, but 3D structure of PAP80-2a and its structure–function relationship is a future challenge.

## Introduction

*Pholiota adiposa*, a fungus that grows from August to October on the dead timber pile of poplars and willows or dead birches, is an edible and medicinal mushroom distributed in China, Korea, Japan, Europe, and North America. It is macro-fungus which belongs to the Strophariaceae family, and is commonly called “yellow-cap fungus or fat mushroom”^1^. The medicinally beneficial functions of *P*. *adiposa* are well known worldwide, including antitumor, antimicrobial, antifatigue, antihypertensive, free radical scavenging, and antihyperlipidemic activities. These medicinal functions are attributing to bioactive polysaccharides, amino acids, and ergosterol in the fruiting bodies or mycelia^2–4^. Especially, polysaccharide is a very important active ingredient in *P*. *adiposa* mycelia. In our previous study, we purified three polysaccharide fractions (PAP30-2b, PAP60-2b, and PAP80-2a) from crude *P. adiposa* polysaccharide (PAP) originating from mycelia. Among these three polysaccharides, PAP80-2a manifested the most prominent antitumor and T-lymphocyte proliferative effects^5^. Therefore, PAP80-2a is a good potential candidate for development of oral drugs for antitumor therapy and immunomodulation. However, the extraction rate of PAP obtained by hot-water-infusion (HWI) approach was extremely low, only 6.89%, which directly led to lower production of PAP80-2a.

Conventionally, polysaccharides from edible mushroom are extracted by hot water procedures. Despite its simplicity and safety, hot water procedures are usually associated with longer extraction time and higher temperature, which may lead to the degradation of the polysaccharides and a decrease of biological activity of target compounds and also lower extraction efficiency of the polysaccharides^6^. Ultrasonic-assisted extraction (UAE) employs ultrasonic waves to effectively facilitate the release of target compounds into the extraction solution, and has attracted much interest because of its innate advantages, including simplified manipulation, significant reduction in energy consumption and higher efficiency^7^. It has proven to be a green and economically viable alternative to conventional extraction techniques for natural products and foods^8,9^. On the other hand, use of an optimization technique such as response surface methodology (RSM) in the extraction processes can be very significative because fewer experimental trails are needed compared to the single-factor method^10^. RSM is widely used for optimizing extraction process variables of bioactive compounds, such as polysaccharides^11^, gelatins^12^, and polyphenols^13^. This technique can identify the interaction between different variables by establishing the appropriate mathematical model.

Our previous work had demonstrated PAP80-2a with excellent antitumor activity^5^. However, its structural features have not been investigated, limiting the application of PAP80-2a. In edible mushrooms, many of extracted polysaccharides are β-linked glucans^14–16^. Structures and functions of polysaccharides extracted from different edible mushrooms are different. It was reported that the polysaccharide isolated from the fruiting bodies of *Dictyophora indusiata*, consisting mainly of (1→3)- β-D-glucan with (1→6)- β-glucosylside branches, had antitumor activities^17^. A water-soluble polysaccharide from Zhuling, having (1→6, 1→4)-linked β-D-glucopyranosyl backbone, was a potent activator of B cells, macrophages and dendritic cells^15^. The chain conformations of polysaccharides are closely related to their functions. Therefore, it is necessary to study the structure of PAP80-2a and its conformations in aqueous solution.

In this study, for the first time, an attempt was made to investigate and optimize the ultrasonic-assisted conditions of polysaccharide from *P*. *adiposa* mycelia in order to enhance the extraction yield of PAP80-2a. Then, an antitumor activity assay of PAP80-2a after ultrasound-assisted extraction was also conducted. In addition, the structural characterizations of PAP80-2a were elucidated by high-performance anion-exchange chromatography with pulsed-amperometric detection (HPAEC-PAD), fourier transform-infrared spectroscopy (FT-IR), nuclear magnetic resonance (NMR), and gas chromatography-mass spectrometer (GC-MS), which could lay the foundation for the development of oral drugs containing this ingredient.

## Results and Discussion

### Single-Factor Experiments

The RSM optimization of the UAE conditions was based on the maximum PAP yield. All the parameters (extraction temperature, extraction time, ultrasonic power, ultrasonic time, and water-to-raw material ratio) were investigated by single-factor experiments in a wide range prior to the RSM optimization. These preliminary experiments enabled the identification of significant factors affecting PAP yield (Y), and narrowed down the ranges for these factors. Finally, Extraction temperature, ultrasonic power and water-to-raw material ratio were confirmed as significant factors that influenced extraction yield.

Temperature, as an independent variable, increases the ability of the solvent to solubilize the compounds and reduces the viscosity of the liquid solvent to allow better penetration of the solvent into the solid matrix^18,19^. The effects of various temperatures (50, 55, 60, 65, 70, 75, 80, 85, 90, 95 and 100 °C) on the extraction efficiency of PAP were investigated by maintaining water-to-raw material ratio and ultrasonic power constant at 30 mL/g and 400 W. PAP yield peaked at 90 °C and then decreased with increasing temperature. Therefore, a temperature range from 85 to 95 °C was used in the RSM experiment to optimize extraction conditions (Supplementary Fig. 1S-A).

Water-to-raw material ratio is an important factor that significantly affects extract yield. If water-to-raw material ratio is too low, polysaccharide in raw material can not be completely extracted. High water-to-raw material ratio result in high process costs. Therefore, a suitable water-to-raw material ratio should be selected for extraction of polysaccharide^20^. Extraction was performed at different water-to-raw material ratio (20, 25, 30, 35, and 40 mL/g) with extraction temperature and ultrasonic power constant at 90 °C and 400 W, respectively. The highest yield of 75 mg/g was reached at a water-to-raw material ratio of 30 mL/g, and there was no obvious increase in PAP yield as the ratio continued to rise. Similar phenomenon was observed in the extraction of *Tricholoma matsutake* polysaccharide. It has been reported that a water-to-raw material ratio rose above 25 ml/g, the polysaccharide yield started to decline slowly^21^. A possible explanation is that the increase of water-to-raw material ratio may increase diffusivity of the solvent into cells and enhance the desorption of the polysaccharide from the cells^22,23^. Thus, water-to-raw material ratio was set in the range of 25–35 mL/g for optimization design (Supplementary Fig. 1S-B).

Different ultrasonic power has been shown to affect the yield of polysaccharide^24^. Thus, the effect of different ultrasonic power on the PAP yield was investigated in this experiment. The ultrasonic power was set in the range of 200 to 1000 W, while the other extraction conditions were fixed at an extraction temperature of 90 °C and a water-to-raw material ratio of 30 mL/g. The extraction yield of PAP was obviously increased as ultrasonic power increased from 200 to 600 W, but slowly increased after 600 W. Ultrasonication facilitated the disruption of cell walls, so the polysaccharides could be dissolved faster. However, extracted liquid became more turbid and centrifugation did not completely separate solid from liquid when the ultrasonic power was higher than 600 W. The turbid solution probably resulted from degradation in polysaccharide structure caused by the high ultrasonic power. Therefore, ultrasonic power of 400–800 W was chosen for RSM optimization (Supplementary Fig. 1S-C).

Based on the preliminary experiments, the RSM experiments were conducted under the following conditions: extraction temperatures range between 85–95 °C, water-to-raw material ratios of 25–35 mL/g and ultrasonic powers of 400–800 W.

### Statistical analysis and model fitting

A total of 17 runs were performed to optimize the three individual parameters, X_1_, X_2_, and X_3,_ using a Box-Behnken design, as shown in Supplementary Table 1S. The maximum yield of PAP (89.47 mg/g) was recorded at an extraction temperature of 90 °C, a water-to-raw material ratio of 30:1, and an ultrasonic power of 600 W.

The fit statistics of extraction yield (Y) for the selected quadratic predictive model is shown in Table 2. The results indicated that the model used to fit the response variable was highly significant (*p* < 0.01) and adequate to represent the relationships between the response and independent variables. Ultrasonic power (X_3_) significantly influenced the extraction yield of PAP (*p* < 0.01), while extraction temperature (X_1_) and water-to-raw material ratio (X_2_) were not significant (*p* > 0.05). In addition, the cross terms (X_1_X_2_, X_1_X_3_, and X_2_X_3_) were not significant, while all the quadratic terms (X_1_, X_2_ and X_3_) significantly affected Y (*p* < 0.01). F-value for the lack of fit was insignificant (*p* > 0.05), thereby confirming the validity of the model. The total determination coefficient (*R*_2_) and adjusted determination coefficient (*R*_adj_) were 0.9852 and 0.9622, respectively, confirming that the model is reasonable and significant.

**Table 1:** Factors and levels.

Factors and levels	Symbol	Factors Symbol Coded levels		
		−1	0	1
Temperature (°C)	X_1_	85	90	95
Water-to-raw material ratio	X_2_	25:1	30:1	35:1
Ultrasonic power (W)	X_3_	400	600	800

**Table 2:** Regression coefficient and significance of each term in the fitted regression model.

Source	Sum of Squares	Freedom degree	Mean Square	F value	P value
Model	648.7082	9	72.07869	51.85195	<0.0001**
X_1_	1.353013	1	1.353013	0.97333	0.3567
X_2_	0.17405	1	0.17405	0.125208	0.7339
X_3_	50.25031	1	50.25031	36.14906	0.0005**
X_1_X_2_	1.452025	1	1.452025	1.044557	0.3408
X_1_X_3_	0.9409	1	0.9409	0.676864	0.4378
X_2_X_3_	2.418025	1	2.418025	1.739478	0.2287
X_1_^2^	125.5685	1	125.5685	90.33144	<0.0001**
X_2_^2^	377.883	1	377.883	271.8413	<0.0001**
X_3_^2^	39.58045	1	39.58045	28.47337	0.0011**
Residual error	9.730605	7	1.390086		
Fit lack	7.394125	3	2.464708	4.219524	0.0990
Pure error	2.33648	4	0.58412		
Total deviation	658.4388	16			

Note: R^2^ = 0.9852, R^2^_Adj_ = 0.9622, CV = 1.96: * means P <0.05 significant difference, **means P <0.01 significant difference.

A second-order polynomial model was generated by applying multiple regression analysis to the experimental data to express the relationship between the independent variables and the response. The final equation obtained in terms of coded factors is given as follows:

$$\begin{array}{c}\begin{array}{rcl}({\rm{Y}}) & = & 88.59-0.41{{\rm{X}}}_{1}+0.15{{\rm{X}}}_{2}+2.51{{\rm{X}}}_{3}+0.60{{\rm{X}}}_{1}{{\rm{X}}}_{2}-0.48{{\rm{X}}}_{1}{{\rm{X}}}_{3}\\  &  & -0.78{{\rm{X}}}_{{\rm{1}}}{{\rm{X}}}_{3}-5.46{{\rm{X}}}_{1}^{2}-9.47{{\rm{X}}}_{2}^{2}-3.07{{\rm{X}}}_{3}^{2}\end{array}\end{array}$$

where Y is the predicted PAP yield, and X_1_, X_2_ and X_3_ are the coded values for extraction temperature, water-to-raw material ratio and ultrasonic power, respectively.

### Analysis of response surfaces, Optimization of extraction parameters and Verification of predictive model

**Figure 1:**
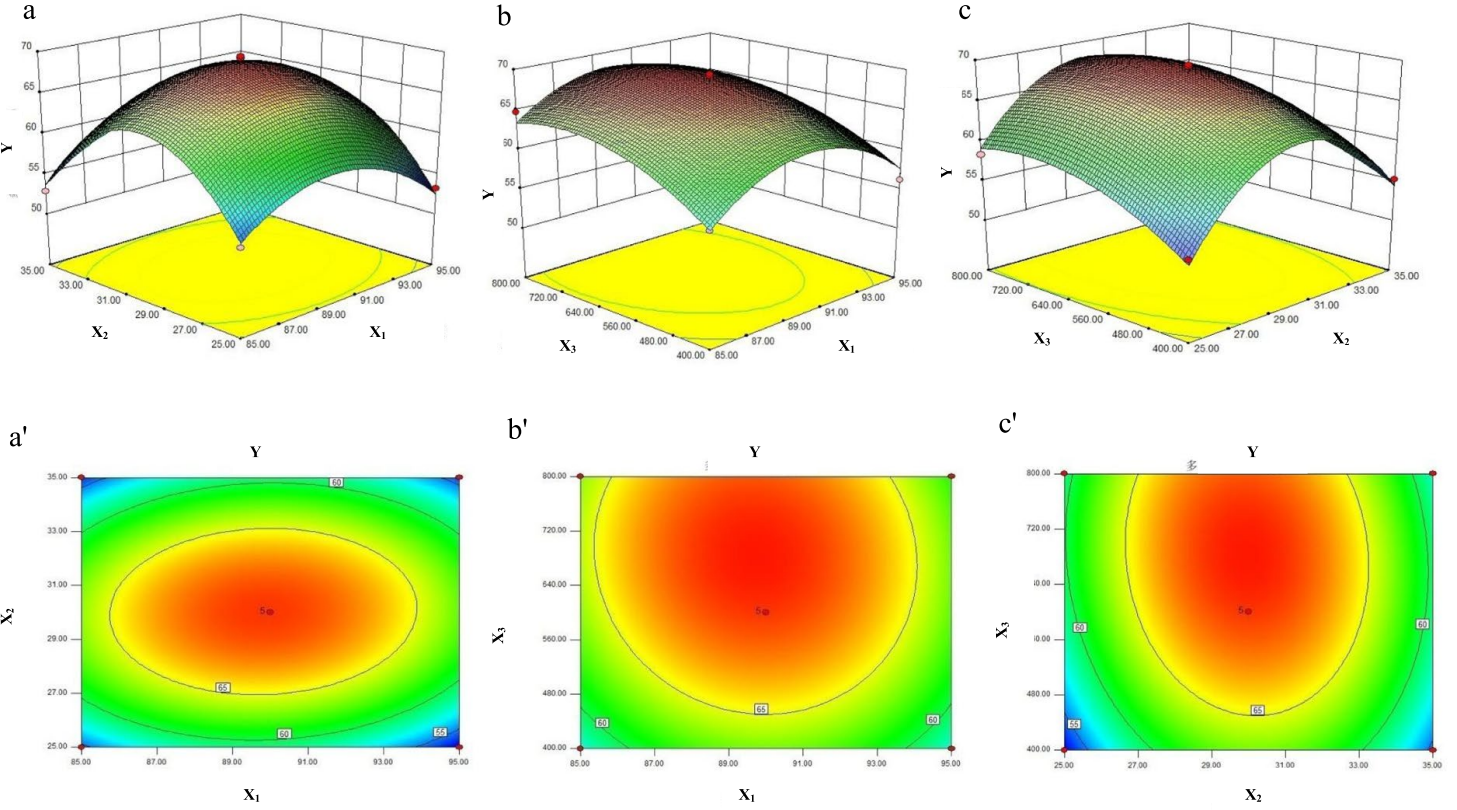
Response surface plots (**a**–**c**) and contour plots (a’-c’) showing the effects of extraction temperature (X_1_), water-to-raw material ratio (X_2_), and ultrasonic power (X_3_) on the extraction yield of PAP.

Three-dimensional (3D) response surface plots and contour plots were generated to visualize the effects of experimental levels of each independent variable on the response and to determine the optimum level of each independent variable for maximum extraction yield of PAP. In each plot, the interaction of two independent variables was investigated simultaneously, while the others were fixed constant at zero level. The 3D response surface plots and contour plots are presented in Fig. 1.

The results of response surface analysis showed that the interaction between extraction temperature and water-to-raw material ratio was significant (Fig. 1a,a’), while that the interaction between extraction temperature and ultrasonic power was insignificant (Fig. 1b,b’). In addition, the influence of ultrasonic power on the peak value of the response was greater than the influence of water-to-raw material ratio (Fig. 1c,c’).

The result of numerical optimization showed that the optimum conditions for PAP yield were as follows: extraction temperature of 89.37 °C, water-to-raw material ratio of 27.65:1, ultrasonic power of 605.15 W. Accordingly, the model predicted a maximum response of 89.51 ± 0.049 mg/g. To ensure the predicted result was not biased toward the practical value, verification experiments were performed by using these modified optimal conditions: extraction temperature of 90 °C, water-to-raw material ratio of 28:1, ultrasonic power of 600 W (Supplementary Table 2S). Triplicate experiments at the modified optimal extraction conditions were carried out to compare the predicted results with the experimental value. A mean yield was 89.88 ± 0.082 mg/g (n = 3), which was close to the predicted value (89.51 ± 0.049 mg/g), but increased by 11.5% compared with that under conventional hot-water-infusion technology (80.61 ± 0.079 mg/g). As a result, central composite design was found to be an accurate and decisive tool for predicting extraction yield of polysaccharides from *P. adiposa* mycelia using an ultrasound-assisted extraction technique.

However, it has been reported that ultrasound might lead to degradation of natural products and thus lead to the loss of bioactivity^25^. In our study, ultrasonic-assisted extraction (UAE) was instead of hot-water-infusion extraction (HWI). It is necessary to determine whether the biological activity of PAP80-2a obtained by ultrasonic extraction still exits. Thus, the antitumor assay in our study focused on the comparision for the antitumor activity of PAP80-2a (obtained by UAE) and PAP80-2a (obtained by HWI).

### Antitumor activity of PAP80-2a

**Figure 2:**
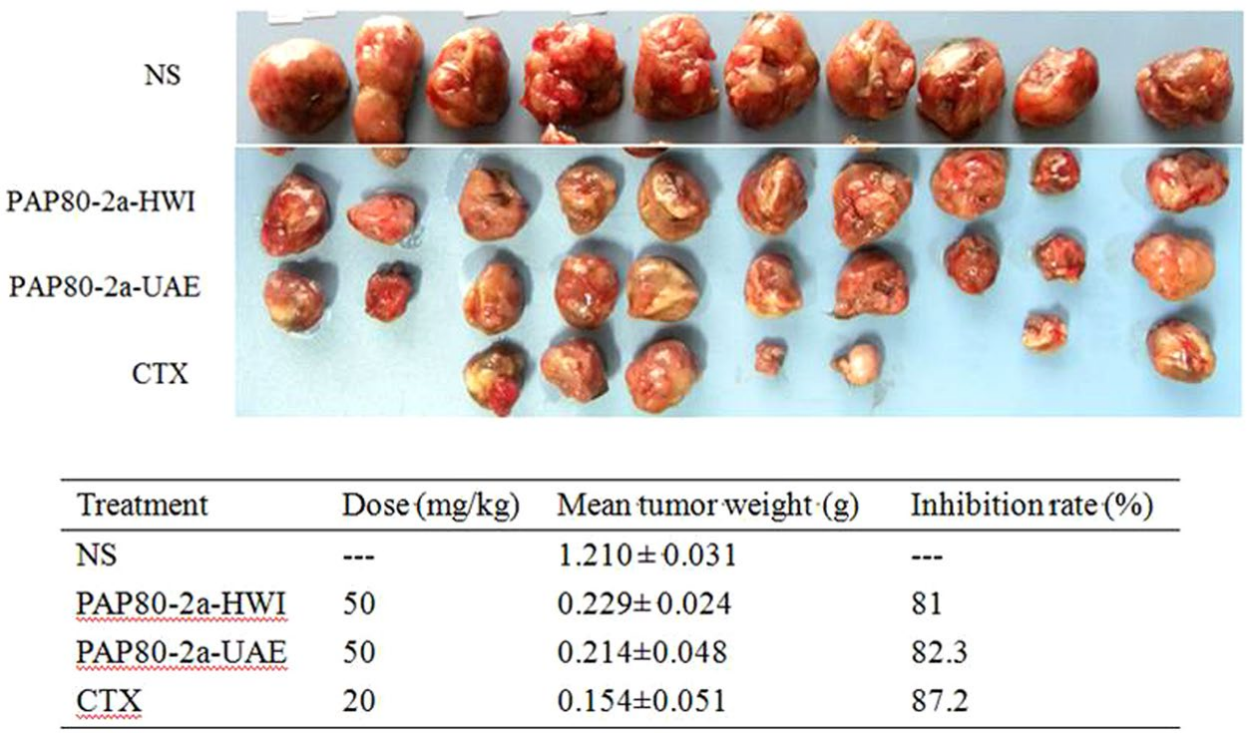
The antitumor activity of PAP80-2a. CTX, NS group were daily intraperitoneally injected with cyclophosphamide or normal saline as positive and negative control, respectively. The doses of PAP80-2a (UAE) and PAP80-2a (HWI) were 50 mg/kg. Ten mice in each group were tested for 2 weeks. Then the weights of tumors were measured. Data are expressed as means of ten determinations ± standard deviation.

Lewis lung cancer cells were subcutaneously injected into C57 mice, and then administrated with normal saline (NS) (20 mL/kg), PAP80-2a (50 mg/kg, UAE), PAP80-2a (50 mg/kg, HWI) and CTX (20 mg/kg) for 2 weeks, respectively. As shown in Fig. 2, the inhibition rate of tumor growth in PAP80-2a (obtained by UAE) group could reach 83.3%, slightly higher than that of PAP80-2a (obtained by HWI) group (81%). It can be seen that the optimized ultrasonic extraction conditions did not destroy the biological activity of PAP80-2a. Although CTX has the most obvious inhibitory effect on tumors growth (87.2%), it had obvious high cell toxicity^5^. A lot of polysaccharides have been extracted from medicinal mushrooms and many of them exhibit antitumor activity^26–28^. Lentinan, a β-1, 3-glucan polysaccharide isolated from *Lentinus edodes*, has been used clinically in Japan since the early 1980s because of its immunomodulatory and antitumor effects^28^. Polysaccharides isolated from *Phellinus linteus* inhibited tumor growth and significantly prolonged the survival of mice with implanted B16F10 melanoma cells^29^.

### Monosaccharide composition analysis of PAP80-2a

**Figure 3:**
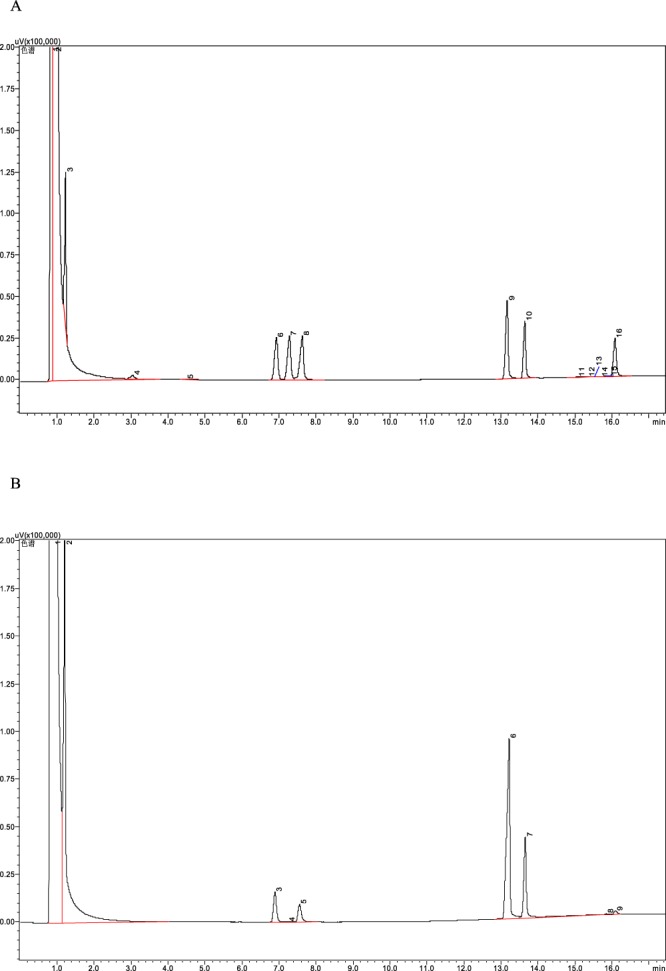
HPAEC analysis of PAP80-2a. (**A**): Typical Chromatography of a standard solution (6 – rhamnose, 7 – arabinose, 8 – xylose, 9 – glucose, 10 - galactose, 16 – mannose). (**B**): HPAEC spectrum of PAP80-2a (3 - rhamnose, 5 - xylose, 6 - glucose, 7 - galactose).

The properties of polysaccharides, such as their composition and molar ratio, make an important contribution to their bioactivity^30^. In order to confirm the structure integrity of PAP80-2a after ultrasonic extraction, we again identify and quantify the monosaccharide composition of PAP80-2a by employing HPAEC-PAD technology. By matching the retention time with those of monosaccharide standards, four peaks were identified in PAP80-2a in the order of rhamnose, xylose, glucose, and galactose with a ratio of 2.09: 1.13: 10.00: 4.09 (Fig. 3). Glucose was the predominant monosaccharide. This result was consistent with our previous report (Seen Hu et. al, 2012).

Antitumor activity and monosaccharide composition analysis of PAP80-2a made us confirm that the ultrasonic-assisted extraction had non-significant influence on the biological activity of PAP80-2a. Based on that conclusion, we further explored the detailed structure characterization of PAP80-2a.

### Amino acid contents in PAP80-2a

The total ion flux of amino acids and the amino acid content of PAP80-2a were measured using the iTRAQ kit in combination with HPLC-MS. As shown in Supplementary Fig. 2S, many amino acids were identified in PAP80-2a. The total content of amino acid in the sugar chain was 69.92 μg/mL. L-aspartic acid content was highest, reaching 10.42 μg/mL, followed by threonine at 7.85 μg/mL, serine at 6.32 μg/mL, glycine at 5.84 μg/mL, and alanine at 5.17 μg/mL (Seen Supplementary Table 3S).

### Glycopeptide linkage form in PAP80-2a

**Figure 4:**
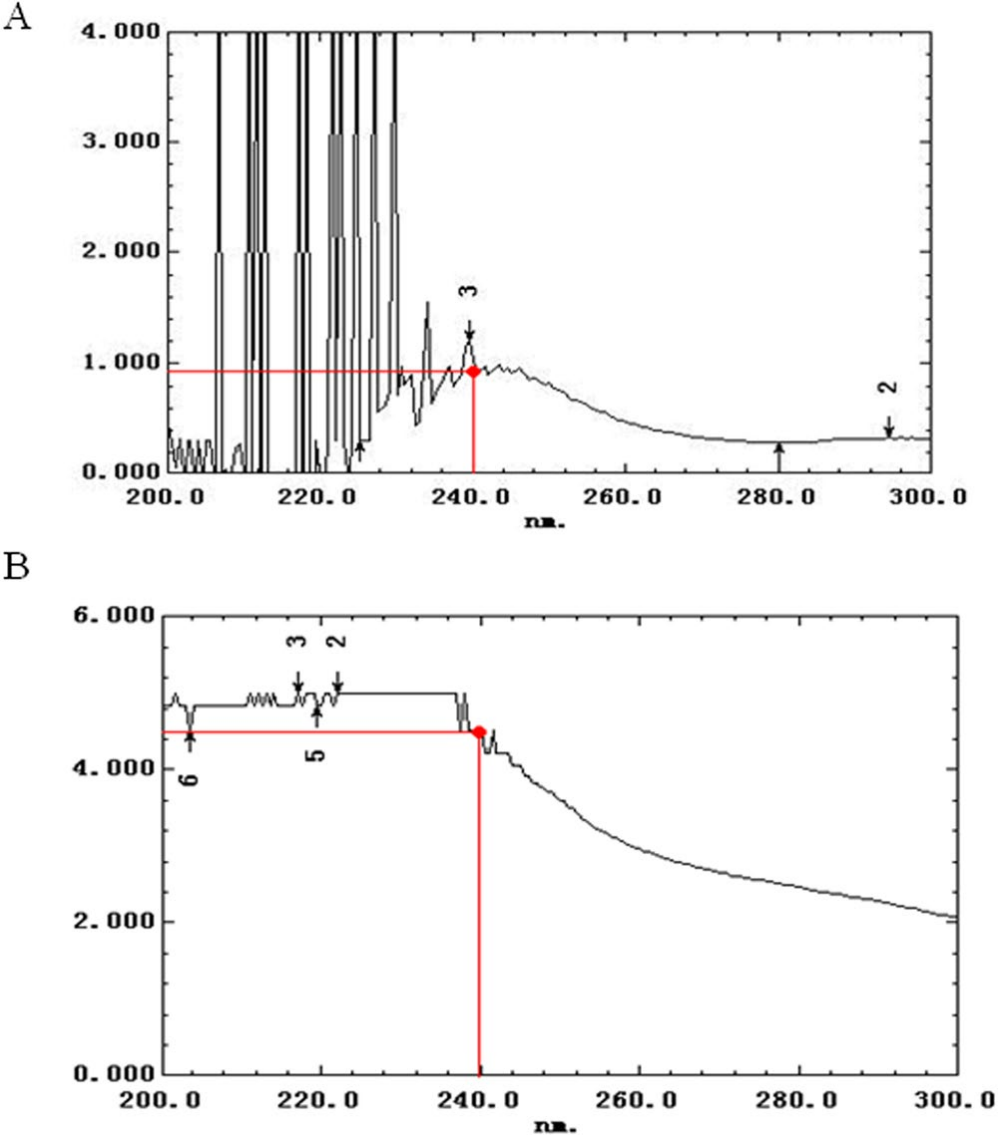
The determination of glycopeptide linkage in PAP80-2a. (**A**) Ultraviolet absorption spectrum before NaOH treatment. (**B**) Ultraviolet absorption spectrum after NaOH treatment.

Alkali treatment may cause the hydrolysis of O-glycopeptide linkages, forming unsaturated amino acids, which can be detected at 240 nm^31^. Ultraviolet absorption spectra before and after NaOH treatment for PAP80-2a are shown in Fig. 4. The absorbance value of glycoprotein before hydrolysis was 0.93 at 240 nm, but this value was significantly enhanced and reached 4.22 after NaOH treatment for 2 h, showing that O-glycopeptide linkages exist in PAP80-2a.

### FT-IR analysis

**Figure 5:**
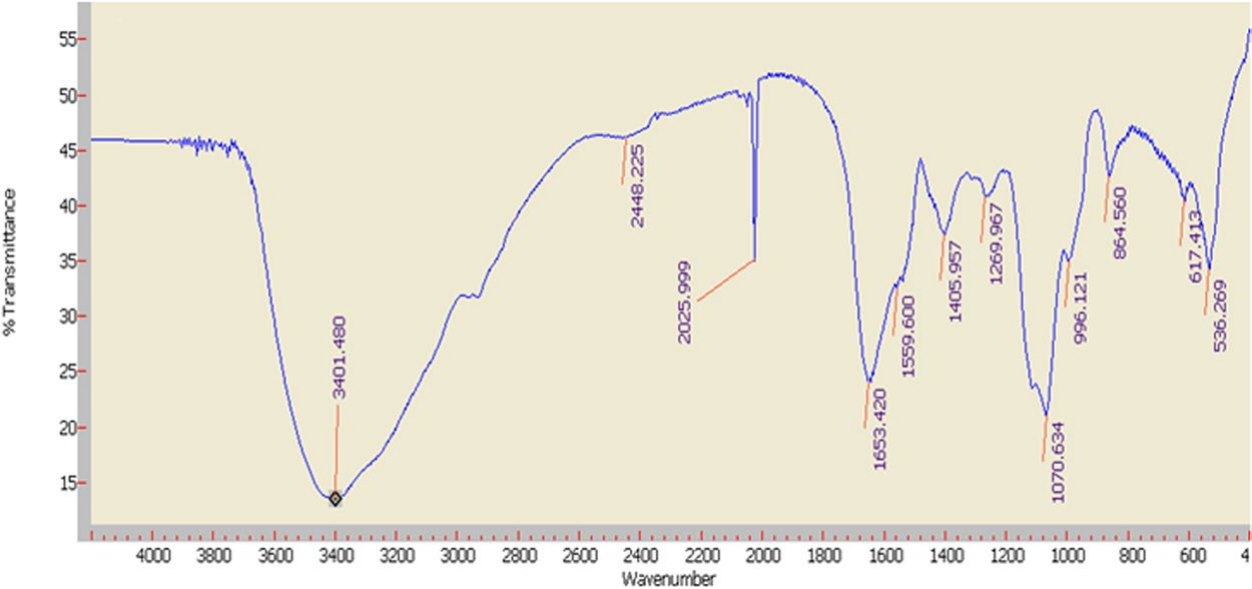
FT-IR spectrum of PAP80-2a at optimum conditions. The horizontal coordinate represents the wave number, and the longitudinal coordinate indicates the absorption peak.

FT-IR spectroscopy can be used for approximate identification of PAP80-2a when combined with chemical analyses. The FT-IR spectrum of PAP80-2a is presented in Fig. 5. The strong and wide stretching peak around 3401.5 cm^−1^ was assigned to O-H stretching vibrations^24^. The signals at 1653.42 and 1269.9 cm^−1^ indicate that an acetyl group (CH_3_-CO-) is present in the polysaccharide chain^32^. An absorbance peak was observed at 864.6 cm^−1^, while no absorbance was present at 891 cm^−1^, suggesting an α-type linkage in PAP80-2a^33^. Strong stretching peaks at 996.1 cm^−1^ and 1070.6 cm^−1^ indicate that pyranoses exist in the α-configuration of PAP80-2a^21^. In addition, we didn’t have any idea on the strong stretching peak at 2025 cm^−1^, and we concluded that this signal may be resulted from some special structure in PAP80-2a or other impurities carried during the purification process of PAP80-2a.

### ^1^H-NMR and ^13^C-NMR analysis

The hydrogen spectrum of PAP80-2a is shown in Supplementary Fig. 3S. Multiple anomeric hydrogen signals were observed in the region between δ 4.5 and 5.5, but no resonance peak with a coupling constant above 6 was found. Therefore, the sugar terminal configuration in the polysaccharide chain is likely an α-type, corresponding to the results obtained by FT-IR analysis.

In the carbon spectra (Supplementary Fig. 4S), the signals in carbon spectrum indicated that the sugar chain contains a number of similar acetyl groups. These acetyl groups may be linked with different sugars or different positions (i.e. in the ring and on the chain) in different sugars. The number and position of the acetyl groups in polysaccharide had a great influence on the activity of polysaccharide^34^. The signal at 102.4 ppm showed an anomeric carbon atom in the polysaccharide structure. Preliminary results, based on the signals at 84.4, 78.7, 75.6, 70.0, and 61.0 ppm, suggested that a [β-Glc (1→2) -]_n_ structure exists in the sugar chain. The preliminary results from the IR spectra indicating an α-glycosidic bond, combined with the signal at 18.5 ppm in the carbon spectrum, suggested that an [α-Rha (1→3) -]_n_ structure also exists in the sugar chain.

### GC-MS analysis

Methylation analysis of the polysaccharide was performed to determine the substitution pattern of monosaccharide residues. The fully methylated sample was converted into partially methylated alditol acetates by hydrolysis, and analyzed by Gas Chromatography-Mass Spectrometry (GC-MS). The presence of 2,4-Me_2_-Glc (15.5%), 3,4-Me_2_-Glc (23.9%), 2,3,4,6-Me_4_-Glc (43.6%), and 2,3,4-Me_3_-Rha (17.0%) showed that 1,3,6-Glc, 1,2,6-Glc, 1-Glc, and 1-Rha are present in the main chain of PAP80-2a (Table 3). Also, we can conclude that rhamnose is located at the non-reducing end of the sugar chain, while the glucose is located at the non-reducing end or in the sugar chain in 1,2,6- and 1,3,6-linked forms.

**Table 3:** Analysis of PAP80-2a by GC-MS.

Sample	t_R_/min	Molar Ratio	Linkage
2, 4-Me_2_-Glc	43.90	15.5	1, 3, 6-Glc
3, 4-Me_2_-Glc	45.75	23.9	1, 2, 6-Glc
2, 3, 4, 6-Me_4_-Glc	24.98	43.6	1-Glc
2, 3, 4-Me_3_-Rha	19.21	17.0	1-Rha

## Conclusion

Ultrasonic-assisted extraction was applied to extract crude PAP from *P. adiposa* mycelia. Response surface methodology along with Box-Behnken design was used to determine the optimal extraction conditions. The optimal extraction parameters are as follows: extraction temperature of 90 °C, water-to-raw material ratio of 28:1, and ultrasonic power of 600 W. PAP yield was increased by 11.15% compared with that under hot-water-infusion technology. This optimization procedure lays the foundation for the further development of food and medicine polysaccharides. In this study, we also determined the structure of PAP80-2a, the main component isolated from PAP. HPAEC-PAD analysis showed that PAP80-2a is mainly composed of glucose, rhamnose, xylose, and galactose in a ratio of 10.00: 2.09: 4.09: 1.13. The ultraviolet absorption method and iTRAQ technology were utilized to demonstrate that an O-glycopeptide linkage exists in PAP80-2a. FT-IR analysis showed that PAP80-2a is a typical α-pyran type polysaccharide. ^1^H-NMR and ^13^C-NMR analysis demonstrated that the sugar chain structure of PAP80-2a is [α-Rha (1→3)-]n, and GC-MS analysis showed that rhamnose is located at the non-reducing end of the sugar chain, while glucose is located at the non-reducing end or in the sugar chain in 1,2,6- and 1,3,6-linked forms. Its structure–function relationship will be the focus of our continuing work.

## Materials and Methods

### Materials

The experimental strain of *Pholiota adiposa*, numbered CAAS2301, was collected from Hebei Province of China by the Mushroom Research and Development Center, Institute of Agricultural Resources and Regional Planning, Chinese Academy of Agriculture Science, and preserved in the China General Microbiological Culture Collection Center (NO.CGMCC1840).

*P. adiposa* was cultured in fermentation medium, composed of glucose (15 g/L), soy peptone (2.5 g/L), KH_2_PO_4_ (0.8 g/L), yeast extract (5 g/L), MgSO_4_·7H_2_O (0.25 g/L), and K_2_HPO_4_ (0.2 g/L), at 27 °C for 9 d, with a shaking speed of 120 rpm. The fermented broth was filtered and centrifuged (8,000 rpm, 10 min), and the precipitate was collected. The filtrate was retained, and mycelia were lyophilized and powdered.

### Ultrasonic-assisted extraction (UAE)

The lyophilized powder of *P. adiposa* mycelia was mixed with a specified amount of distilled water. The extraction process was performed using an ultrasonic instrument (JY99-IIIBN) with a 3.00 mm flat tip probe according to the experimental design. The extracting solution was centrifuged at 8000 rpm for 15 min and the supernatant was concentrated with a rotary evaporator. The concentrated supernatant was treated with the Sevag reagent three times to remove protein^35^ and then mixed with four volumes of 95% ethanol and kept overnight at 4 °C. The precipitates were collected by centrifugation (5000 rpm for 20 min), washed three times with ethanol and acetone to obtain the crude extract. Finally, the washed precipitates were dried at 40 °C until its weight was constant. The polysaccharide extraction yield was calculated as follows:

$$\begin{array}{rcl}{\rm{Extraction}}\,{\rm{yield}}( \% ) & = & \mathrm{100}\times \mathrm{weight}\,{\rm{of}}\,{\rm{dried}}\,{\rm{crude}}\,{\rm{polysaccharide}}({\rm{mg}})\\  &  & /\mathrm{weight}\,{\rm{of}}\,{\rm{mycelia}}\,{\rm{powders}}({\rm{g}})\end{array}$$

### Experimental Design, Optimization and Validation of Optimized Conditions

The UAE conditions for PAP were optimized through central composite response surface methodology (RSM). On the basis of preliminary results (data not shown), proper ranges of three independent variables including extraction temperature (X_1_), water-to-raw material ratio (X_2_) and ultrasonic power (X_3_) were obtained and categorized into three levels, coded +1, 0, and −1 for high, intermediate, and low value, respectively (Table 1). The whole design was composed of 17 experimental runs carried out in random order, with six replications at the center point to estimate the repeatability of the method. The data were analyzed by multiple regression analysis using Design-Expert 8.0 and then the polynomial equation to represent PAP yield as a function of the independent variables tested was derived:

The significance of all the terms in the model was assessed by the probability (*p*) of 0.05. The adequacy of the model was checked accounting for determination coefficient (*R*^2^), adjusted determination coefficient (*R*^2^_adj_), the coefficient of variance (CV) and predicted error of sum of square.

Numerical optimization was carried out to predict the exact optimum level of independent variables leading to the desirable response goal. In this regard, all the independent variables were kept within range, while the response was maximized. Additional triplicate experiments were carried out under optimal extraction conditions in order to determine the validity of optimized conditions. The average value of the validation experiment was compared with the predicted value of the developed condition in order to find out the accuracy and suitability of the optimized conditions.

### The purification of PAP80-2a from PAP

Under the optimal conditions, PAP was obtained from *P. adiposa* mycelia. A stepwise ethanol precipitation assay was used to fractionate the PAP. Briefly, 95% ethanol was slowly added to the supernatant to final alcohol concentrations of 60% and 80%. Accordingly, two purified fractions termed PAP60 and PAP80 were obtained. PAP80 was dissolved in 50 mL distilled water and subsequently loaded on a column of DEAE-Sepharose Fast Flow column (26 mm × 100 cm) by an AKTA Explorer (Sweden). The active fraction PAP80-2a was obtained by elution with 0-2 M NaCl solution.

### Animals and ethical statement

Male inbred mice line C57 (6–8 weeks old, weight 20 ± 2 g) was purchased from Beijing Likeaidai Counseling Service (certificate No. scxk2011-0001). Animals were housed in plastic cages and environmental conditions were kept constant, in agreement with the standards for animal housing.

All experimental protocols were approved by the Institutional Safety Office and Animal Experimentation Ethics Committee at Chinese Academy of Agricultural Sciences. All the methods were carried out in accordance with the approved guidelines of the Animal Care and Use Committee of Chinese Academy of Agricultural Sciences.

### Tumor inhibition assay for PAP80-2a

This assay was carried out according to our previous report^5^. C57 mice were subcutaneously injected at the armpit with 5 × 10^6^ Lewis lung cancer cells in 0.2 mL, and randomly assigned to four groups (normal saline group, cyclophosphamide group, and PAP80-2a group (UAE) and PAP80-2a group (HWI)). There were 10 mice in each group and the experiment was repeated three times under identical conditions. All mice in each group were injected intraperitoneally daily with normal saline (NS) (20 mL/kg), PAP80-2a (50 mg/kg, HWI), PAP80-2a (50 mg/kg, UAE) and cyclophosphamide (CTX, 20 mg/kg), respectively. Ten normal mice (control group) received no injections. After 2 weeks of administration, all mice were sacrificed by cervical dislocation, and the body weight, tumors weight were measured. The effectiveness of each sample was evaluated by the inhibition rate, which was calculated by the equation Inhibition rate (%) = (C – T)/C × 100%, where C means tumor weight in control group (g) and T means tumor weight in test groups (g).

### Monosaccharide composition analysis of PAP80-2a

Monosaccharide analysis was performed as described by Chen, *et al*.^21^ with some modifications. PAP80-2a (2 mg) was added to an eggplant shaped bottle, and 3 mL of trifluoroacetic acid (2 mol/L) was added. The mixture was hydrolyzed at 110 °C for 3 h, cooled to room temperature, and dried under vacuum at 40 °C. Methanol (3 mL) was added to the bottle after drying, and drying was continued under vacuum. Redistilled water was then added to a constant volume of 100 mL. Nine monosaccharides, D-Gal, D-Glc, D-Ara, L-Fuc, L-Rha, D-Man, D-Xyl, D-GluA, and D-GalA, were used as standards. The sample (25 μL) was analyzed using high-performance anion-exchange chromatography with pulsed-amperometric detection (HPAEC-PAD) and a CarboPac^TM^PA20 column (3 mm × 150 mm). The temperature of the column was set at 30 °C, and the flowing phase was 0.25 M NaOH and 1 M NaAc. Monosaccharide identification was performed by comparison with a reference monosaccharide. The relative molar ratio is calculated by the area normalization method. For reference, the following neutral sugars were converted to their acetylated derivatives and analyzed: D-Glc, D-Gal, D-Ara, L-Rha, D-Man, L-Fuc and D-xyl.

### Amino acid composition analysis of PAP80-2a

HCl (500 μL, 6 mol/l) was added to 10 mg of sample, and the sample was hydrolyzed at 110 °C for 22 h while sealed. Hydrolyzed sample (250 μL) was mixed with 750 μL of methanol solution. The mixture (100 μL) was dried under nitrogen, then dissolved in methanol. Before performing HPLC-MS analysis, the sample was derived according to the iTRAQ kit introductions.

### Determination of glycopeptide linkage in PAP80-2a

The linkage structure in PAP80-2a was determined using an ultraviolet absorption method as follows. Sample (5 mg) was dissolved in 3 mL of distilled water and then divided into two duplicate samples. One sample was added to 1.5 mL of distilled water, and subjected to spectrum scanning at 200-300 nm. The other sample was added to 1.5 mL of NaOH (0.4 mol/L), and subjected to spectrum scanning (240 nm wavelength) at the beginning and end of the reaction (2 h).

### Fourier transform infrared (FT-IR) analysis

The FT-IR spectrum of the polysaccharide sample was recorded with a Shimadzu-9A FT-IR spectrometer (Shimadzu Company, Japan) using the potassium bromide (KBr) disks method. Briefly, the sample was dried at 35–44 °C with vacuum over P_2_O_5_ for 48 h, ground with KBr powder, and then pressed into pellets for FT-IR spectral measurement in the frequency range of 400–4000 cm^−1^.

### Nuclear Magnetic Resonance (NMR) analysis

NMR spectra (^1^H NMR and ^13^C NMR) were recorded on a Bruker-ARX-600 spectrometer (Rheinstetten, Germany) using tetra-methyl silane (TMS) as an internal standard and D_2_O as the solvent at 25 °C.

### Gas Chromatography-Mass Spectrometry (GC-MS) assay

Methylation of the sample was performed before GC-MS assay. Methylation steps were as follows. The PAP80-2a sample (8 mg) was dissolved in 2.5 mL dimethyl sulfoxide (DMSO) in a 15 mL three-necked flask. After 20 mg of dried NaOH power was added, the mixture was agitated for 30 min with a magnetic stirrer under the protection of N_2_. Methyl iodide (0.3 mL) was added to the reaction system under the same conditions. The reaction was stopped by adding 0.5 mL of distilled water, and the reaction solution was dialyzed against running water for 48 h and distilled water for 24 h. The dialyzed product was freeze-dried to obtain a partially methylated sample. Completely methylated sample was obtained by repeating the methylation procedures several times. The completely methylated sample (5 mg) was hydrolyzed with 2 mL of 2 M trifluoroacetic acid and acetylated as described above. The acetylated derivative was filtered through a 0.22 µm nylon membrane and analyzed by GC-MS (Satum 2200 GC-MS, Varian) with a UA-5 quartz capillary column (DB-5MS, 30 m × 0.25 mm × 0.25 µm). The temperature program was set as follows: the initial temperature of the column was set at 80 °C and held for 1 min, then increased to 210 °C at 8 °C /min and held for 1 min, then increased to 260 °C at 20 °C /min and maintained for 1 min. The flow rate was 1 ml/min. The injection temperature was 250 °C. The ion source was set at 250 °C. Range of mass charge ratio (m/z) was set at 40–500 amu. Scan time was set at 0.28 s.

### Statistical analyses

Statistical analysis was performed using Design-Expert (Version8.0, Stat-Ease) and SPSS (Version 17.0) statistical software. Two groups were compared using Student’s t-test. For multiple comparisons, one-way analysis of variance (ANOVA) was used.

## Supplementary information

supplementary data

## Data Availability

All data included in this study are available upon request by contact with the corresponding author.
