# Supplementary material for: The structural characterization of a polysaccharide exhibiting antitumor effect from Pholiota adiposa mycelia
Source: Sci Rep. 2019 Feb 11;9:1724. doi: 10.1038/s41598-018-38251-6 (PMC6370848; doi:10.1038/s41598-018-38251-6)
Supplement: Supplementary file 1 — supplementary data [file 41598_2018_38251_MOESM1_ESM.pdf]

The structural characterization of a polysaccharide exhibiting antitumor effects from *Pholiota adiposa* mycelia

Yajie Zou[1], Fang Du[1], Qingxiu Hu[1*], Hexiang Wang[2]

[1]Institute of Agricultural Resources and Regional Planning, Chinese Academy of Agricultural Sciences, 12 Zhongguancun South Street, Beijing 100081, China

[2]State Key Laboratory for Agrobiotechnology and Department of Microbiology, China Agricultural University, 2 Yuanmingyuan West road, Beijing, 100193, China

*Corresponding author: Tel (Fax): +86 10 82108681

E-mail: Huqingxiu@caas.cn (Qingxiu Hu)

Yajie Zou and Fang Du contributed equally to this work.

Table 1S The experimental design and results for response surface analysis

Runs	X_1	X_2	X_3	PAP yield（mg/g）
1	-1	0	-1	77.45
2	-1	0	1	84.82
3	1	-1	0	73.25
4	-1	-1	0	73.96
5	0	0	0	87.97
6	-1	1	0	72.86
7	0	-1	-1	73.22
8	0	1	1	77.33
9	1	1	0	74.56
10	0	0	0	88.54
11	0	1	-1	75.26
12	0	-1	1	78.40
13	0	0	0	87.73
14	0	0	0	89.25
15	1	0	-1	76.28
16	1	0	1	81.71
17	0	0	0	89.47

Note: X_1、X_2 and X_3 are temperature, material-water ratio, and ultrasonic power, respectively.

Table 2S Predicted and experimental values of the responses at optimum and modified conditions

	Extraction temperature (℃)	Water-to-raw material ratio	Ultrasonic power (W)	PAP Yield (mg/g)
Optimum condition	89.37	27.65:1	605.15	89.51
Modified condition	90	28:1	600	89.88

Table 3S Amino acids content of PAP80-2a

Amino acid	Molecular	Concentration ($\mu g.mL^{-1}$)
Taurine	$C_2H_7NSO_3$	0.08
L-serine	$C_3H_7NO_3$	6.32
Hydroxy-L-proline	$C_5H_9NO_3$	0.48
Glycine	$C_2H_5NO_2$	5.84
L-glutamine	$C_5H_{10}N_2O_3$	0.54
L-aspartic acid	$C_4H_7NO_4$	10.42
Ethanolamine	C_2H_7NO	0.09
L-histidine	$C_{11}H_{17}N_3O_4$	1.78
L-threonine	$C_4H_9NO_3$	7.85
β-alanine	$C_3H_7NO_2$	0.94
L-alanine	$C_3H_7NO_2$	5.17
L-glutamic acid	$C_5H_9NO_4$	5.35
L-arginine	$C_6H_{14}N_4O_2$	2.76
L-α-amino-adipic acid	$C_6H_{11}NO_4$	0.07
γ-amino-n-butyric acid	$C_4H_9NO_2$	0.08
D, L-β-amino-isobutyric	$C_4H_9NO_2$	0.06
L-proline	$C_5H_9NO_2$	3.66
L-ornithine	$C_5H_{12}N_2O_2$	1.18
L-cysteine	$C_3H_7NO_2S$	1.82
L-lysine	$C_6H_{14}N_2O_2$	3.94
L-methionine	$C_5H_{11}O_2NS$	1.64
L-valine	$C_5H_{11}NO_2$	2.96
L-tyrosine	$C_9H_{11}NO_3$	0.59
L-isoleucine	$C_6H_{13}NO_2$	1.83
L-leucine	$C_6H_{13}NO_2$	2.50
L-phenylalanine	$C_9H_{11}NO_2$	1.94
Total content ($\mu g.mL^{-1}$)		69.92

Figure 1S. Effect of extraction temperature (A), water-to-raw material ratio (B), and ultrasonic power (C) on the extraction yield of PAP.

Figure 2S. Ion chromatogram of amino acid contents of PAP80-2a.

Figure 3S. The ^{1}H-NMR spectrum of PAP80-2a. The horizontal coordinate represents the value of chemical shift.

Figure 4S. The ^{13}C-NMR spectrum of PAP80-2a. The horizontal coordinate represents the value of chemical shift.

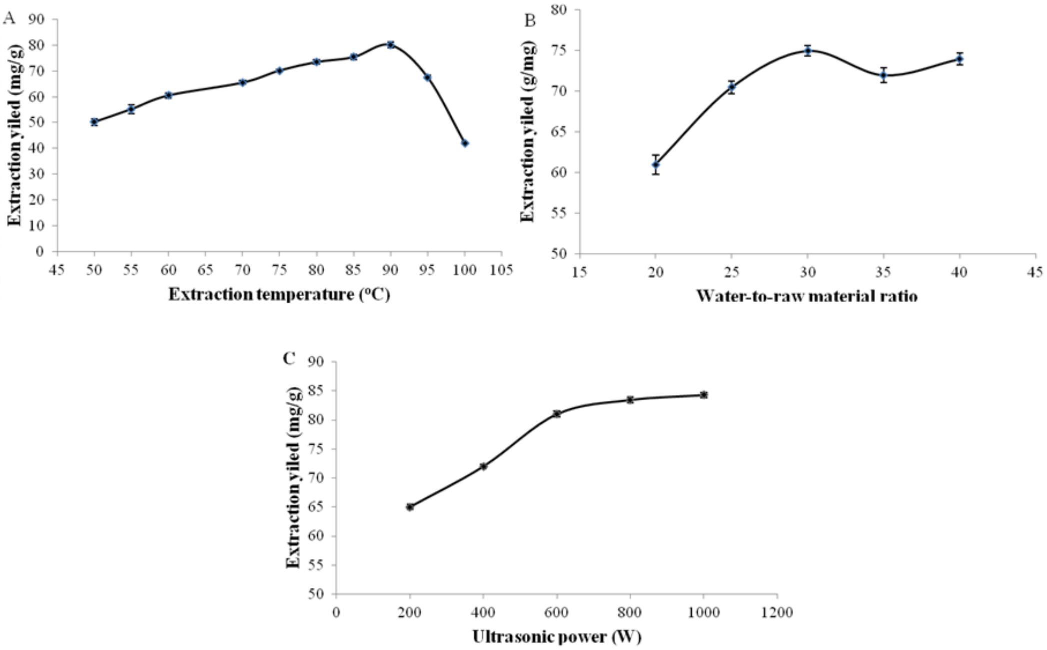
A
Extraction yiled (mg/g)
Extraction temperature (ºC)
B
Extraction yiled (g/mg)
Water-to-raw material ratio
C
Extraction yiled (mg/g)
Ultrasonic power (W)

Fig. 1S

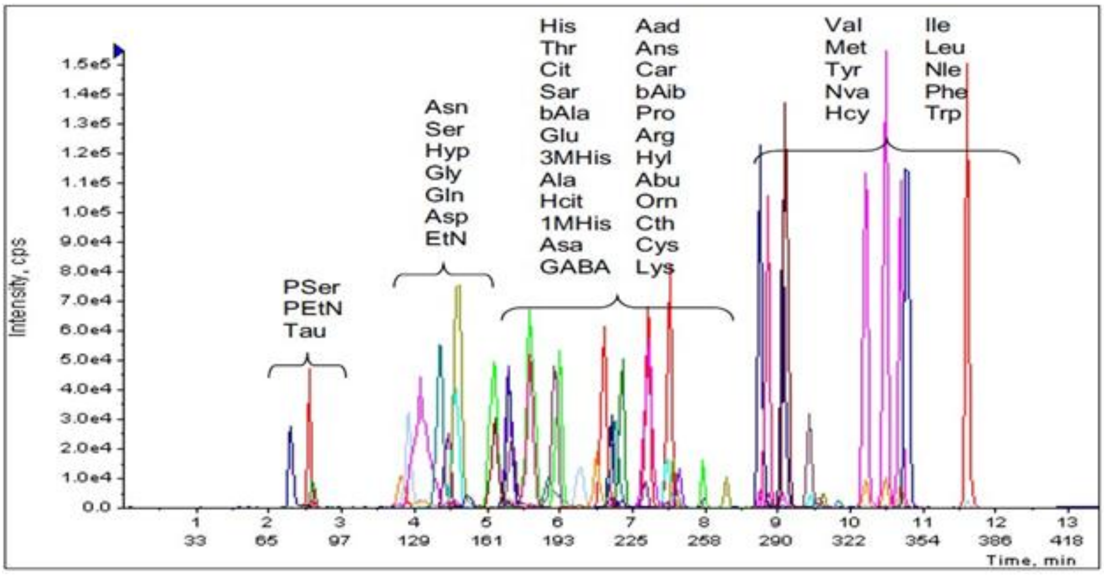
Intensity, cps
1.5e5
1.4e5
1.3e5
1.2e5
1.1e5
1.0e5
9.0e4
8.0e4
7.0e4
6.0e4
5.0e4
4.0e4
3.0e4
2.0e4
1.0e4
0.0
PSer
PEtN
Tau
Asn
Ser
Hyp
Gly
Gln
Asp
EtN
His
Thr
Cit
Sar
bAla
Glu
3MHis
Ala
Hcit
1MHis
Asa
GABA
Aad
Ans
Car
bAib
Pro
Arg
Hyl
Abu
Orn
Cth
Cys
Lys
Val
Met
Tyr
Nva
Hcy
Ile
Leu
Nle
Phe
Trp
1 2 3 4 5 6 7 8 9 10 11 12 13
33 65 97 129 161 193 225 258 290 322 354 386 418
Time, min

Fig. 2S

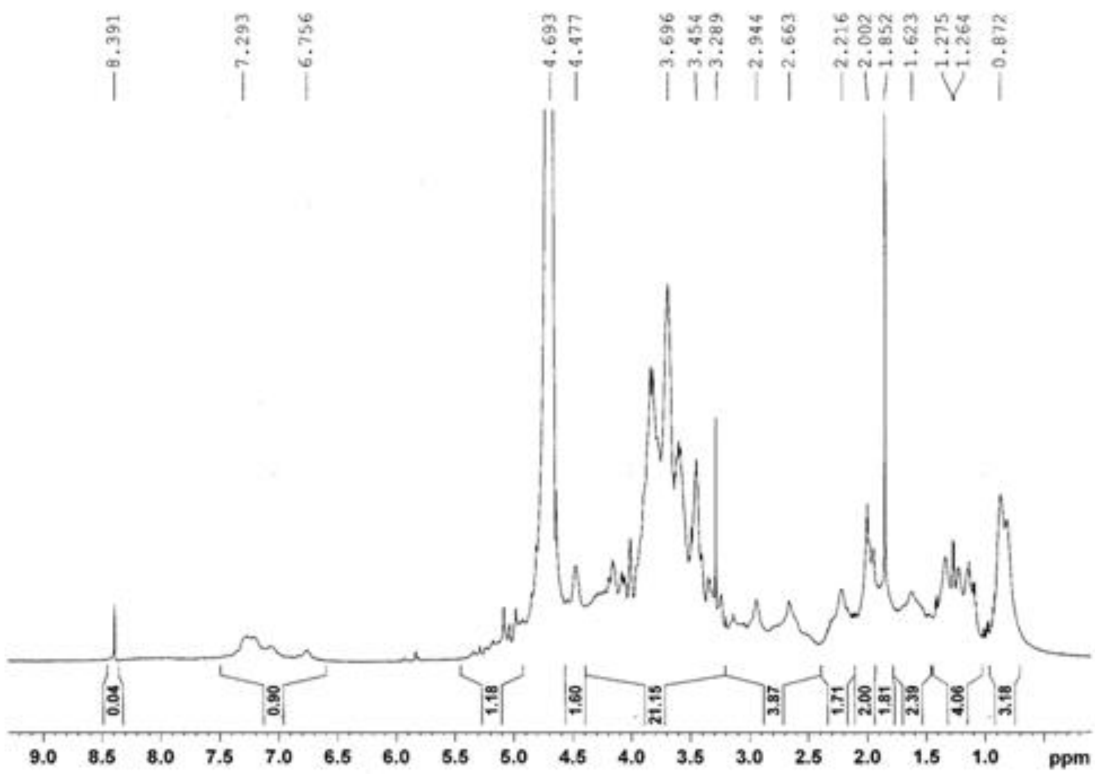
8.391
7.293
6.756
4.693
4.477
3.696
3.454
3.289
2.944
2.663
2.216
2.002
1.852
1.623
1.275
1.264
0.872
0.04
0.90
1.18
1.60
21.15
3.87
1.71
2.00
1.81
2.39
4.06
3.18
9.0
8.5
8.0
7.5
7.0
6.5
6.0
5.5
5.0
4.5
4.0
3.5
3.0
2.5
2.0
1.5
1.0
ppm

Fig. 3S

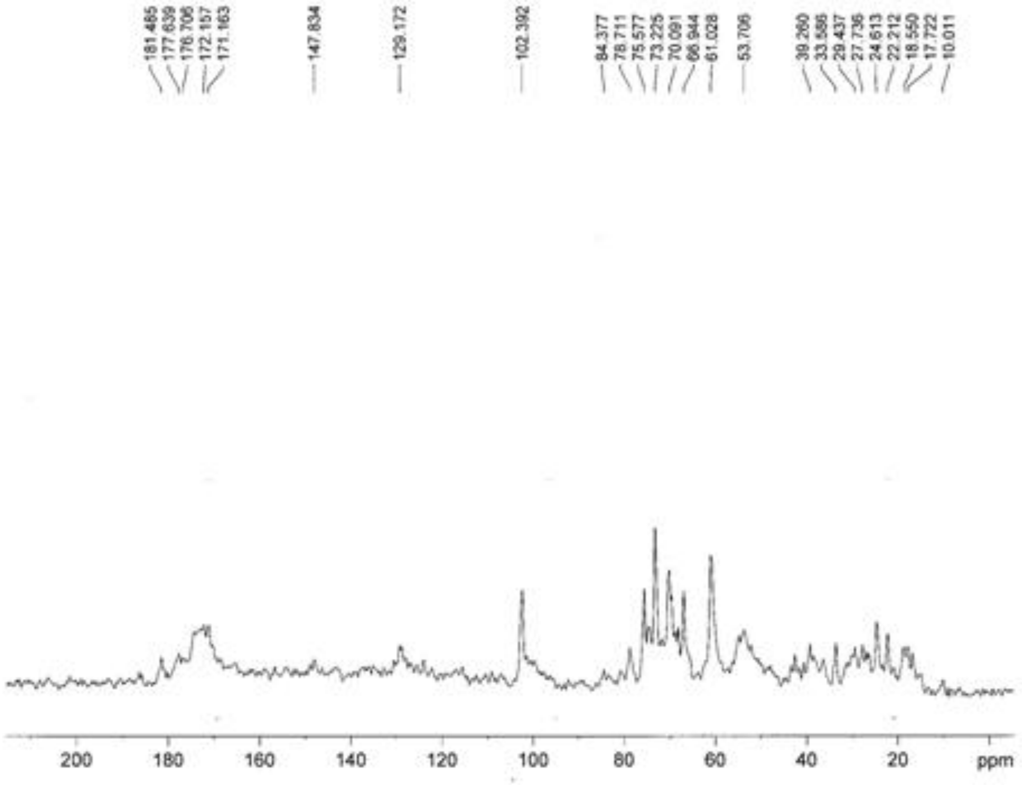
181.465
177.639
176.706
172.157
171.163
147.834
129.172
102.392
84.377
78.711
75.577
73.225
70.091
66.944
61.028
53.706
39.260
33.586
29.437
27.736
24.613
22.212
18.550
17.722
10.011
200
180
160
140
120
100
80
60
40
20
ppm

Fig. 4S
